# The application of CRISPR/Cas9 system in cervical carcinogenesis

**DOI:** 10.1038/s41417-021-00366-w

**Published:** 2021-08-04

**Authors:** Chun Gao, Ping Wu, Lan Yu, Liting Liu, Hong Liu, Xiangyu Tan, Liming Wang, Xiaoyuan Huang, Hui Wang

**Affiliations:** 1 Cancer Biology Research Center (Key laboratory of the ministry of education), Tongji Hospital, Tongji Medical College, Huazhong University of Science and Technology, Wuhan, Hubei China; 2 Department of Gynecologic Oncology, Sun Yat-Sen University Cancer Center, State Key Laboratory of Oncology in South China, Collaborative Innovation Center for Cancer Medicine, Guangzhou, China; 3 Department of Gynecologic Oncology, Tongji Hospital, Tongji Medical College, Huazhong University of Science and Technology, Wuhan, Hubei China; 4 Department of Gynecologic Oncology, Women’s Hospital, School of Medicine, Zhejiang University, Zhejiang, China

**Keywords:** Genetic engineering, Cervical cancer

## Abstract

Integration of high-risk HPV genomes into cellular chromatin has been confirmed to promote cervical carcinogenesis, with HPV16 being the most prevalent high-risk type. Herein, we evaluated the therapeutic effect of the CRISPR/Cas9 system in cervical carcinogenesis, especially for cervical precancerous lesions. In cervical cancer/pre-cancer cell lines, we transfected the HPV16 E7 targeted CRISPR/Cas9, TALEN, ZFN plasmids, respectively. Compared to previous established ZFN and TALEN systems, CRISPR/Cas9 has shown comparable efficiency and specificity in inhibiting cell growth and colony formation and inducing apoptosis in cervical cancer/pre-cancer cell lines, which seemed to be more pronounced in the S12 cell line derived from the low-grade cervical lesion. Furthermore, in xenograft formation assays, CRISPR/Cas9 inhibited tumor formation of the S12 cell line in vivo and affected the corresponding protein expression. In the K14-HPV16 transgenic mice model of HPV-driven spontaneous cervical carcinogenesis, cervical application of CRISPR/Cas9 treatment caused mutations of the E7 gene and restored the expression of RB, E2F1, and CDK2, thereby reversing the cervical carcinogenesis phenotype. In this study, we have demonstrated that CRISPR/Cas9 targeting HPV16 E7 could effectively revert the HPV-related cervical carcinogenesis in vitro, as well as in K14-HPV16 transgenic mice, which has shown great potential in clinical treatment for cervical precancerous lesions.

## Introduction

Cervical cancer is the fourth most common malignancy in women worldwide [1]. Human papillomavirus 16 (HPV16) is the most predominant high-risk HPV type with the highest risk of progression to cervical malignancy [2, 3]. More than 80% of women who have at least one opposite-sex partner will acquire HPV infection in their lifetime [4]. High-risk HPV persistent infection has been considered to be a leading cause of cervical carcinogenesis [5]. Researchers also found that the HPV viruses could integrate their genes into the human genome, and it seemed to be a critical event in the progression of carcinogenesis [6]. The integration of HPV leads to persistent expression of the HPV oncogene, making it difficult to eliminate. At present, there is no effective treatment for patients with persistent HPV infection or the integration of HPV genes [7].

The gene-editing tools mainly include zinc finger nuclease (ZFN) [8], transcription activator-like effector nuclease (TALEN), and clustered regularly interspaced short palindromic repeat (CRISPR/Cas9). All these gene-editing tools could induce targeted DNA double-strand breaks (DSBs) and edit targeted genes by stimulating the DNA repair mechanisms [9]. With the improvements of these gene-editing tools, gene therapy is becoming more precise and effective. In previous studies, our team has demonstrated that these gene-editing techniques designed for HPV oncoprotein genes could effectively influence targeted cells [10, 11]. However, the comparison of the efficacy of gene therapies in HPV infection disease is not identified yet.

For the CRISPR/Cas9 system, researchers only need to design the gRNA complementary to the target DNA sequence, without any other component [8]. The CRISPR/Cas9 system might be an ideal alternative to ZFN and TALEN for inducing targeted gene editing because it is rapid and easy to design. It could cause DSB at the specific site which could be fixed by the cells’ self-repairing system in the form of NHEJ (non-homologous end-joining) or HDR (homologous-dependent repair), resulting in gene deletion, reversion, and insertion [9]. Some previous reports have suggested that the CRISPR/Cas9 system targeted HPV oncogene might have a therapeutic effect on HPV-related cervical cancer [12]. However, there has been a scarcity of dynamic observation of the treatment process in a suitable animal model until now.

The high-risk HPV oncogenes E6 and E7 play a key role in the development of carcinogenesis through the interaction with tumor suppressor genes—E6 for TP53 and RB for E7 [13, 14]. HPV 16 E6 targeting TP53 could induce infected cells to cease apoptosis and the transformed cells were able to continually replicate E7 oncoprotein binds to the retinoblastoma family members for degradation, resulting in the release of E2F transcription factors which indirectly promote the replicate of transformed cells [15, 16]. A recent study showed that during the infectious period, the HPV16 genome shared more amino acid-changing variants, while E7 was genetically strictly conserved [17]. This meant that the gene-editing tool targeting the HPV16 E7 oncogene had more clinical significance in the therapy of cervical carcinogenesis.

In this study, we conducted CRISPR/Cas9 targeting HPV16 E7 in vitro and in vivo experiments and compared its efficiency with ZFN and TALEN. The knockout of the E7 oncogene-induced cell apoptosis and reduced cell proliferation. In vivo experiment, we have experimentally demonstrated the therapeutic effect of the CRISPR/Cas9 system by utilizing K14-HPV16 mice, which integrated the HPV16 gene and could formation of cervical pre-cancer lesion spontaneously. Our results indicated the great potential of the CRISPR/Cas9 system in clinical treatment.

## Materials and methods

### Cell culture and transfection

The cervical cancer cell lines SiHa, HeLa, C33A were purchased from the American Type Culture Collection (ATCC) and cultured in Dulbecco’s modified Eagle’s medium (DMEM) supplemented with 10% fetal bovine serum (FBS, Gibco) and 100 U/ml of penicillin and streptomycin (Invitrogen) at 37 °C in a humidified incubator with 5% CO_2_. The S12 cell line was a gift from Pro. Kenneth Raj (Health Protection Agency), and it was permitted by the original owner, Pro. Margaret Stanley. The S12 cells which contained the integrated HPV16 genome was an immortalized human cervical keratinocyte cell line and were cultured in a mixture of DMEM and Ham F12 medium at a ratio of 1:3, which was supplemented with 5% FBS, 8.4 ng/ml of cholera toxin, 5 μg/ml of insulin, 24,3 μg/ml of adenine, 0.5 μg/ml of hydrocortisone and 10 ng/ml of EGF. All the cells were transfected by X-tremeGENE HP DNA Transfection Reagent (Roche) according to the manufacturer’s instructions. The ratio of reagent to DNA was optimized in preliminary experiments.

### Plasmid

The gRNA targeting HPV16 E7 was designed using the online tool (http://crispor.tefor.net/crispor.py) according to the protocol of Mali et al. [18] in our lab and synthesized by the Genewiz Company [19]. The Cas9 plasmid was obtained from Addgene. The sgRNA sequence targeting HPV16 E7 was provided from our previous study [20]. The sequence of gRNA-HPV16 E7-1 was 5’-GCTGGACAAGCAGAACCGGA-3’, and the sequence of gRNA-HPV16 E7-2 was 5’-GAGACAACTGATCTCTACTG-3’. The ZFN (MA13 and MA14) and TALEN (T512) plasmids were from our own laboratory, which was used in primary experiments [10, 11]. We cloned the sgRNAs into the pSpCas9(BB)-2A-GFP (#48138) obtained from Addgene.

### T7E1 assay

After transfection for 48 h, the DNA of cells was extracted using the QIAamp DNA Mini kit. The primers used for amplification are F: tgtcaaaagccactgtgtcc, R: taaaatctaccaaatcttcacctgt, 200 ng purified polymerase chain reaction (PCR) products containing the sgRNA targets were denatured and reannealed. 2 units of T7E1 enzyme (NEB) were added to the tube and incubated at 37 °C for 30 min. The digested products were viewed in 2% agarose gels, and the gene-editing rate was calculated as follows: (1-(1-cleaved bands)1/2) × 100. Each experiment was repeated 3 times.

### Western blot analysis

After 48 h of transfection of the CRISPR plasmid, the protein of the cells was extracted and quantified. Forty micrograms of total proteins were used in the 1% SDS-PAGE electrophoresis. The primary antibodies used were rabbit anti-HPV16-E7 (1:200, orb10573, Biorbyt), rabbit anti-RB (1:1,000,10048-2-Ig, Proteintech), rabbit anti-CDK2 (1:200,10122-1-AP, Proteintech), rabbit anti-E2F1 (1:200, 12171-1-AP, Proteintech), rabbit anti-GAPDH (1:5,000, 60004-1-Ig, Proteintech). The experiment was repeated 3 times.

### Immunohistochemistry (IHC) and immunofluorescence staining

The xenografts in nude mice and the uterine cervixes and vaginas of the transgenic mice were isolated and fixed by 4% paraformaldehyde. Paraffin-embedded sections (5 μm) were subjected to IHC staining according to the Proteintech protocol (http://www.ptgcn.com/support/protocols/). After antigen retrieval for 30 min and blocking with 3% hydrogen peroxide for 20 min, the paraffin sections were incubated overnight at 4 °C by the following primary antibodies: rabbit anti-HPV16E7 (1:100, orb10573, Biorbyt), rabbit anti-RB (1:200,10048-2-Ig, Proteintech), rabbit anti-CDK2 (1:200, ab6538, Abcam), rabbit anti-E2F1 (1:200, 12171-1-AP, Proteintech), rabbit anti-Ki67 (1:200, ab16667, Abcam), rabbit anti-p16 (1:100, A11337, Abclonal), rabbit anti-PCNA (1:100, 10205-2-AP, Proteintech), rabbit anti-CD31 (1:100, 11265-1-AP, Proteintech), rabbit anti-Caspase-3 (1:100, 19677-1-AP, Proteintech). Next, samples were incubated with proper secondary antibodies for 1 h at room temperature. A 3,3’-diaminobenzidine (DAB) kit was used to detect the antibodies, and the slide was photographed at the random site using the cellSens Dimension (version 1.8.1, Olympus). The staining intensity was measured by ImagePro Plus.

### Cell proliferation assay

Cell proliferation was determined using Cell Counting Kit-8 (CCK8) according to the manufacturer’s instructions. After 24 h of transfection of CRISPR/Cas9 plasmids, cell lines, including S12, SiHa, C33A, and HeLa, were seeded in a 96-well plate with 2 × 10^3^ per well. Ten microliters of CCK8 dye and 90 μl fresh DMEM was added to each well, and the cells were incubated for 3 h. The absorbance at 450 nm was read by a microplate reader.

### Apoptosis assay

After 48 h transfection of CRISPR/Cas9 plasmids, SiHa, C33A, HeLa, and S12 cells were collected and washed 3 times in PBS. Annexin V-FITC apoptosis kits (KenGen Biotech) were used following the manufacturer’s protocol, and samples were detected on the FACS Calibur^TM^ (BD Bioscience). The experiments were repeated 3 times.

### Colony-forming assay

Twelve hours post-transfection, SiHa and S12 cells were digested and washed with PBS, and 200 cells per group were plated into the 6-well plate. After 14-day culture, the cells were stained with 4% crystal violet and scanned. The colony numbers were counted using ImageJ.

### Animal experiments

All animal experiments were approved by the Ethical Committee of Tongji Hospital, Tongji Medical College, Huazhong University of Science and Technology. 16 four-week-old Balb/c-nu female mice (bodyweight 15–16 g) were purchased from Beijing HFK Biotechnology Co., Ltd. and kept at the Experimental Animal Center, Tongji Medical College, HUST. 5 × 10^6^ S12 cells were resuspended in 100 μl of 1× PBS and injected subcutaneously in the right flank of Balb/c-nu mice. When the xenografts reached approximately 100 mm^3^, the mice were randomly assigned to 4 groups, with 4 mice in each group. A mixture of 10 μg of plasmid and TurboFect in vivo Transfection Reagent (#R0541, Thermo Fisher Scientific) were injected intratumorally every 3 days according to the manufacturer’s protocol. The volume of the xenografts was measured and recorded using a digital Vernier caliper every 6 days. Mice were sacrificed 5 weeks after SiHa cell injection, and the tumors were surgically isolated and weighed.

FVB.Cg-Tg(KRT-HPV16)wt1Dh (K14-HPV16) transgenic mice were provided by the National Cancer Institute (NCI) Mouse Repository (Frederick, Maryland, USA). The mice were also housed and bred at the Experimental Animal Center, Tongji Medical College, HUST. The genotyping of the offspring has been described elsewhere in detail. The female K14-HPV16 mice were randomly assigned to different groups when they were 6–8 weeks old. After anesthetization, 10 μg of plasmids complexed with TurboFect were piped into the vagina of the mice, which was washed 3 times with saline. The mice were kept on the electric heating blanket for at least 30 min dorsally. The vagina and other organs were dissected and fixed for HE or IHC.

### Statistical analysis

All statistical analysis was performed on SPSS 21.0 (SPSS Inc., Chicago, IL, USA) and GraphPad Prism 8 (GraphPad Software, USA). The significance of different groups was determined by a 2-tail student’s *t*-test. The results are expressed by mean (±) standard deviation (SD). *p*-values below 0.05 were considered statistically significant.

## Results

### CRISPR/Cas9 system efficiently mediates cleavage of the HPV16 E7 gene in HPV16-positive cells compared to ZFN and TELAN

**Fig. 1:**
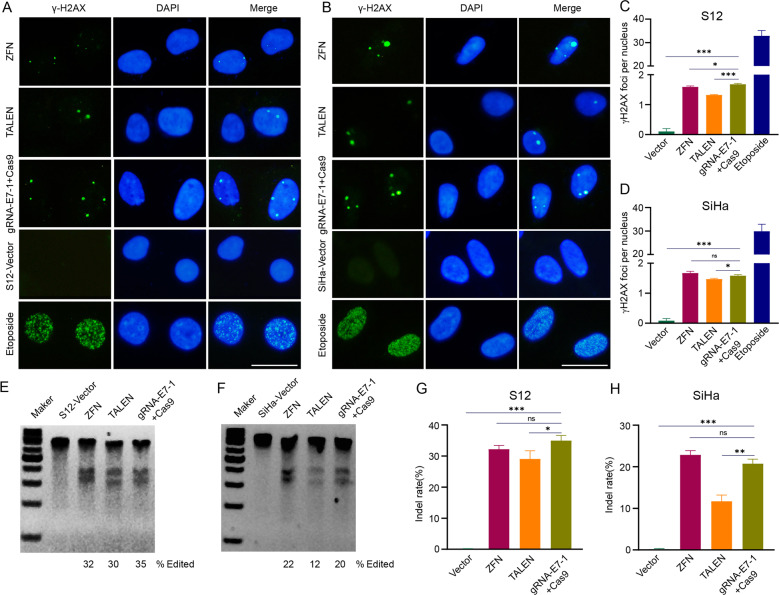
Efficacies comparison of ZFN, TALEN, and CRISPR/Cas9 on HPV16-positive cell lines. The representative images of γ-H2AX foci (green signals) in ZFN-treated, TALEN-treated, and CRISPR/Cas9-treated S12 cells (**A**) and SiHa cells (**B**). Etoposide (0.25 μM) was used as the positive control, and vector plasmid was used as the negative control. Quantification of γ-H2AX foci in S12 (**C**) and SiHa (**D**). T7 endonuclease 1 (T7E1) assay of ZFN-induced, TALEN-induced, and CRISPR/Cas9-induced cleavage at 48 h in S12 cells (**E**) and SiHa cells (**F**). Quantification of DNA indel rate in S12 (**G**) and SiHa (**H**). ns, no significance; *p < 0.05; **p < 0.01; ***p < 0.001. (*n* = 3 replications) Scale bars: 50 μm. Each experiment was repeated 3 times.

To compare the ability of specifically induced DNA cleavage among CRISPR/Ca9, ZFN, and TELAN in HPV16 positive cell lines, we used the immunofluorescence (IF) staining of γ-H2AX to detect DSBs. We separately expressed the CRISPR/Cas9, TALEN, and ZFN plasmids in HPV16-positive cancer cell line SiHa and HPV16-positive immortalized cervical epithelial cell line S12. Previous research reported that there were 1–2 copy numbers and 1–3 copy numbers of HPV16 among the SiHa cells and S12 cells, respectively [10]. After treatment, the number of γ-H2AX foci was increased from 0.1 ± 0.1 (Vector) to 1.67 ± 0.03 (CRISPR/Ca9), 1.59 ± 0.04 (ZFN), and 1.32 ± 0.02 (TALEN) per nucleus in SiHa, and from 0.08 ± 0.07 (Vector) to 1.58 ± 0.04 (CRISPR/Ca9), 1.67 ± 0.06 (ZFN), and 1.46 ± 0.03 (TALEN) per nucleus in S12 (*p* < 0.05, compared with Vector) (Fig. 1A–D).

We used a mismatch-sensitive T7 endonuclease I (T7EI) assay to validate the targeted DNA disruption (Fig. 1E–H), and the results of T7EI digestion proved that there were corresponding indel mutations in the HPV16 E7 gene region of cell lines. In the S12 cell line, the frequency of the CRISPR/Cas9-induced indel mutations had the highest efficiency among the 3 tools. In the SiHa cell line, the CRISPR/Cas9 seemed to be more effective than TALEN in mediating DNA disruption, which might show differences in 3 gene-editing techniques among the different cancer cell lines. The T7E1 assay could only detect the mismatch sequences of genes. However, it did not have access to validate identical mutant sequences. Hence, the real efficiency of gene editing techniques inducing DNA mutation may be underestimated.

### Knockdown of HPV16 E7 induced apoptosis of specific HPV-positive cells

**Fig. 2:**
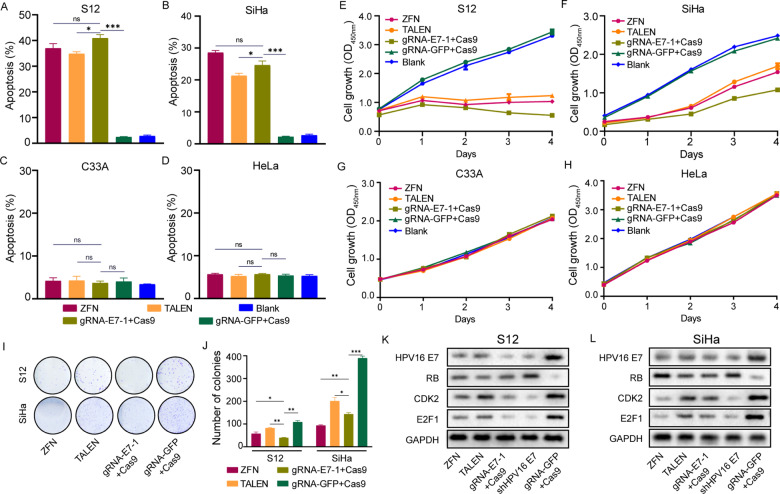
ZFN, TALEN, and CRISPR/Cas9 induced cell growth deficit and cell apoptosis in vitro. **A**–**D** Growth curves of ZFN, TALEN, and CRISPR/Cas9-treated S12 (**A**), SiHa (**B**), C33A (**C**), and HeLa (**D**) cells were constructed using the CCK-8 assay. **E**–**H** The apoptosis rate of S12 (**E**), SiHa (**F**), C33A (**G**), and HeLa (**H**) cells 48 h after treatment with ZFN, TALEN, and gRNA-E7-1 + Cas9 plasmids. **I** The colony-forming assay of SiHa and S12 cells after treatment with ZFN, TALEN, and gRNA-E7-1 + Cas9 plasmids. **J** Quantification of the number of colonies in S12 and SiHa cells of different treatment groups. **K**–**L** HPV16 E7/RB/CDK2/E2F1 expression of S12 (**K**) and SiHa (**L**) cells 48 h after treatment with ZFN, TALEN, and gRNA-E7-1 + Cas9 plasmids. ns, no significance; *p < 0.05; **p < 0.01; ***p < 0.001. (*n* = 3 replications) The experiments were repeated 3 times.

To investigate the cell apoptosis induced by gene-editing techniques, each 1 × 10^6^ cells (SiHa, S12, HeLa, and C33A) were transfected with the corresponding plasmid. We found increased apoptotic fractions by CRISPR/Cas9 among HPV16-positive cell lines SiHa and S12. However, in HeLa and C33A cell lines, the marginal effects of apoptosis were observed. A similar phenomenon of cell apoptosis was also found in ZFN and TALEN conducted HPV16-positive cell lines (Fig. 2A–D).

### CRISPR/Cas9 mediates specific inhibition of cell growth and colony formation of HPV16 positive cells

To explore whether the decrease of E7 expression could affect the growth of HPV16-positive cells, we transfected CRISPR/Cas9-E7 plasmid into HPV16-positive cell lines SiHa and S12 and used HPV18-positive cell line HeLa and HPV-negative cell line C33A as the control to detect the specific effect of CRISPR/Cas9 on cell growth by CCK8 assay (Fig. 2E–H). The growth of SiHa and S12 cell lines was significantly inhibited for 4 days. However, there was no significant difference between CRISPR/Cas9-treated groups and control groups among the C33A and HeLa cell lines shown in Fig. 2C–D.

Furthermore, we used the colony-forming assay to investigate the possible negative effect of CRISPR/Cas9 on the specific HPV-positive cells (Fig. 2I–J). After transfecting the CRISPR/Cas9 plasmid, we found that the colony formation number of S12 and SiHa cells significantly decreased after 2 weeks, and similar results were obtained in the TALEN-treated and ZFN-treated groups.

### CRISPR/Cas9 reduces the expression of HPV16 E7 and recovers the expression of the related protein in HPV16-positive cell lines

The results of the T7E1 assay and γ-H2AX confirmed that the CRISPR/Cas9 system could successfully induce HPV16 E7 gene cleavage. To compare the ability of specifically induced decreased expression of HPV16 E7 oncoprotein, we transfected CRISPR/Cas9, ZFN, and TALEN in HPV16-positive cell lines. shRNA HPV16 E7 was used as a positive control, and gRNA-GFP + Cas9 was used as a negative control. After 48 h, as observed by Western blotting, the CRISPR/Cas9 system could efficiently reduce the HPV16 E7 expression in SiHa and S12 cells (Fig. 2K–L). In the S12 cell line, CRISPR/Cas9 was observed to more effectively decrease E7 expression than the other two gene-editing tools. In addition, we found that the HPV16 E7 related protein expressions have also be influenced. With the decrease of HPV16 E7 expression, the expression of RB protein was increased. At the same time, downstream proteins of RB, E2F1, and CDK2, were downregulated.

### CRISPR/Cas9 inhibits tumor formation in vivo and affects the expression of the corresponding protein

**Fig. 3:**
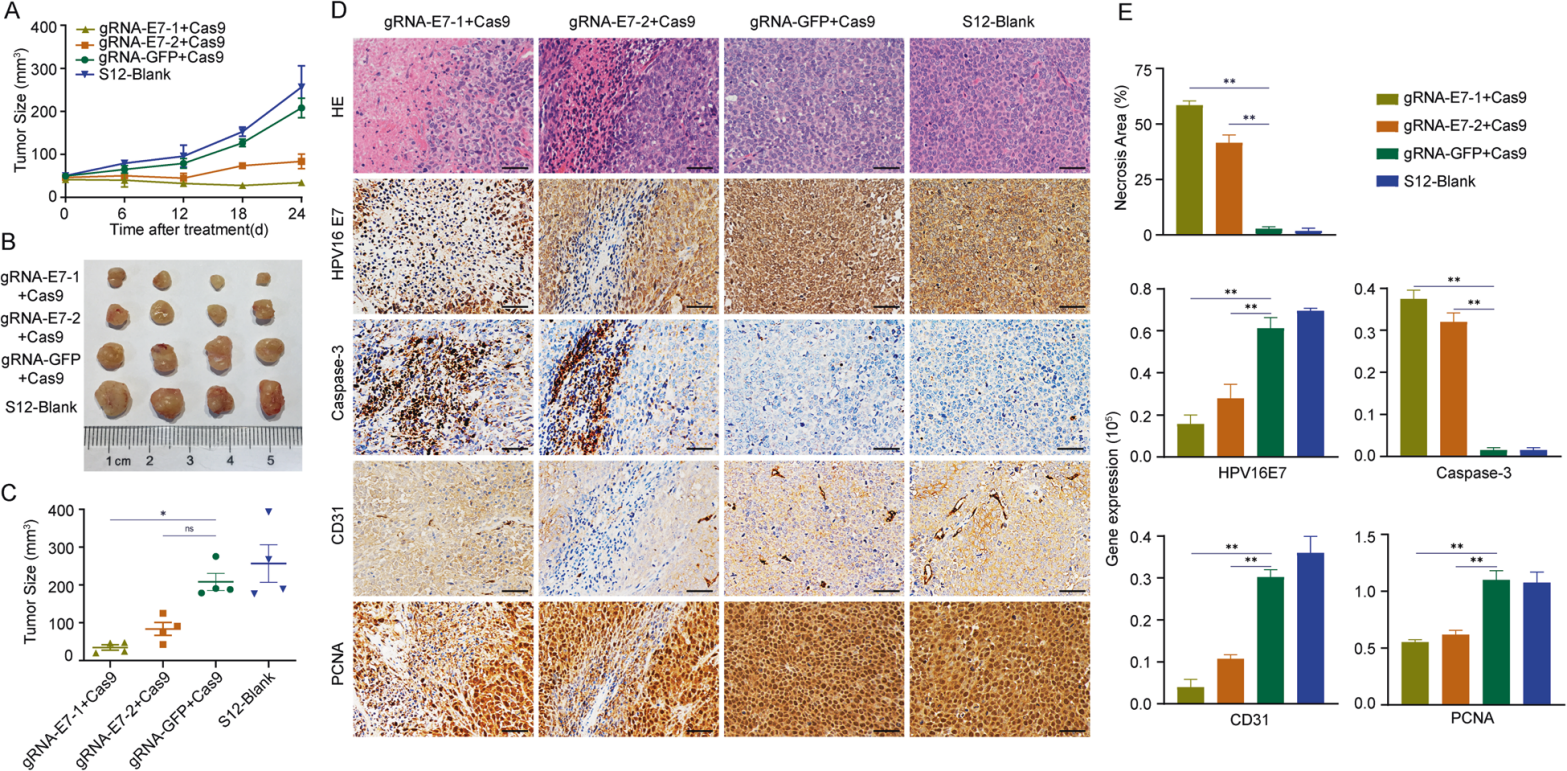
CRISPR/Cas9 inhibits S12 cell growth in vivo. Balb/c-nu mice were injected subcutaneously in the right flanks with 5 × 10^6^ of S12 cells. Then, the CRISPR/Cas9 plasmids complexed with in vivo transfection reagents were injected intratumorally when the xenografts reached approximately 50 mm^3^. **A** The xenografts were measured every 6 days after treatment with gRNA-E7-1 + Cas9, gRNA-E7-2 + Cas9, gRNA-GFP + Cas9, and PBS. **B** The photograph of S12 xenografts in different treatment groups. **C** The estimated tumor size of S12 xenografts in different treatment groups. **D** Representative pictures of HE staining and IHC staining of HPV16 E7, Caspase-3, CD31, and PCNA in gRNA-E7-1 + Cas9, gRNA-E7-2 + Cas9, gRNA-GFP + Cas9, and PBS treated S12 xenografts. Scale bars: 50 μm. **E** The average necrosis area and protein expression of HPV16 E7, Caspase-3, CD31, and PCNA in different groups. **p < 0.01. (*n* = 4 replications).

To further explore the effect of CRISPR/Cas9-introduced inhibition of tumorigenicity in vivo, we inoculated S12 cells in Balb/c nude mice subcutaneously to form xenografts models. We injected the CRISPR/Cas9 plasmid into the tumor using transfection reagent and measured the size of xenografts every 6 days, and we used the gRNA-GFP + Cas9-treated group as the vector group. During 24 days, we found that the size of the tumors of the 2 CRISPR/Cas9-treated groups (gRNA-E7-1 + Cas9/ gRNA-E7-2 + Cas9) were significantly smaller, and tumors that formed grew more slowly compared to the blank group and vector group (Fig. 3A–C). There was a statistical difference in the tumor size between the gRNA-E7-1 group and the vector group. Next, we performed hematoxylin and eosin (H&E) staining and immunohistochemistry staining on the xenograft tumor sections with HPV16 E7, Caspase-3, CD31, and PCNA antibodies. Compared with blank and vector groups, the 2 CRISPR-treated groups had higher expression of Caspase-3 and lower expression of HPV E7, CD31, and PCNA (Fig. 3D–E).

### Vaginal application of CRISPR/Cas9 induces E7 mutation and the reversal of cervical malignancy in K14-HPV16 transgenic mice

**Fig. 4:**
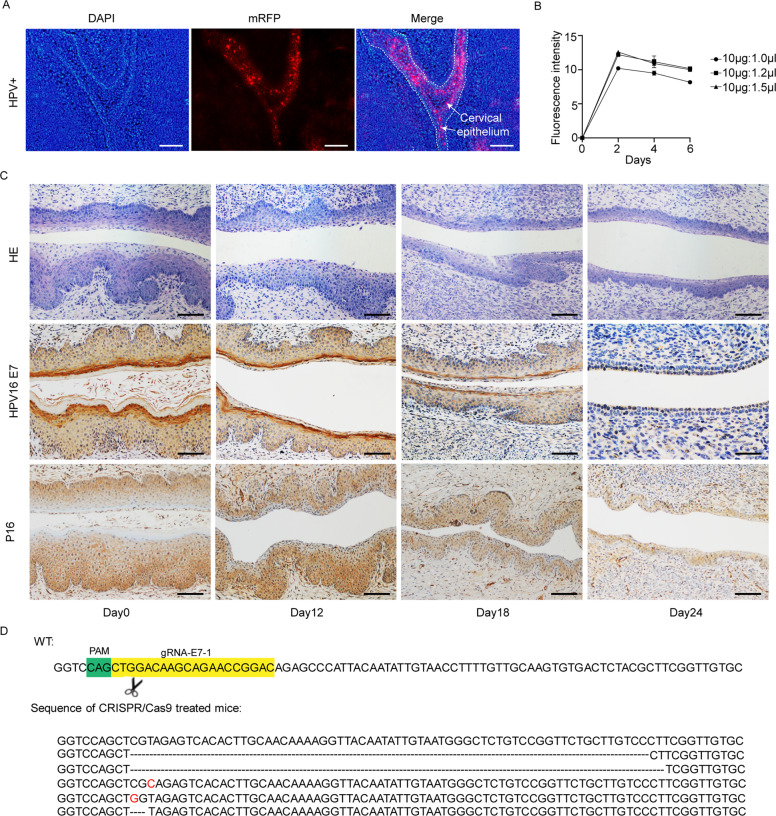
Establishment and application of CRISPR/Cas9 system in K14-HPV16 transgenic mice. **A** The expression of mRFP was localized in the cervical epithelia of transgenic mice. Scale bars: 50 μm. **B** Transfection efficiency was optimized at the DNA-to-polymer ratio of 10 μg:1.0 μl, 10 μg:1.2 μl, and 10 μg:1.5 μl. The exfoliation of cervical cells was collected at 2, 4, and 6 days after vaginal transfection. **C** Representative HE staining and IHC staining of HPV16 E7 and p16 of gRNA-E7-1 + Cas9 treated K14-HPV16 transgenic mice at days 0,12,18, and 24. *N* = 3, Scale bars: 50 μm. **D** The cervical DNA sequencing of the gRNA-E7-1 targeted region of HPV16 E7 gene in gRNA-E7-1 + Cas9 treated K14-HPV16 transgenic mice.

We introduced the HPV16 integrated mice model to further explore the efficacy and dynamic changes in the treatment of the CRISPR/Cas9 system in vivo. The K14-HPV16 mice could spontaneously exhibit different degrees of squamous epithelial hyperplasia in the cervical cervix and vagina, which could be an ideal animal model for evaluating the therapeutic efficacy. We evaluated the mRFP plasmid to prove the successful expression of the plasmid in the mouse vagina. This showed that the mRFP fluorescence could last at least 6 days. Six days after transfection of mRFP plasmids, the vagina of K14-HPV16 mice was extracted, and the red fluorescence was observed in the frozen section (Fig. 4A). To explore the most rational ratio, we tested different plasmid to polymer ratios in mice and decided the highest transfection efficiency ratio by comparing the mRFP expression in exfoliated cervical cells after transfection (Fig. 4B). Finally, we chose a DNA-to-polymer ratio of 10 μg:1.2 μl for the following experiments.

To observe the dynamic changes during the therapy, we applied the vaginal transfection of gRNA-E7-1 + Cas9 in mice every 3 days and sacrificed mice at different time points. After 12 days, we found that the expression of HPV16 E7 protein was decreased in cervical epithelia in IHC results. This trend became increasingly obvious over time, and HPV16 E7 expression was almost invisible on the 24th day. At the same time, we also observed the decreased expression of P16 protein in cervical epithelial cells. The H&E results showed that in gRNA-E7-1 + Cas9 treated mice the CIN of mice was gradually returned to normal-like cervical epithelial with the increase of treatment time (Fig. 4C).

**Fig. 5:**
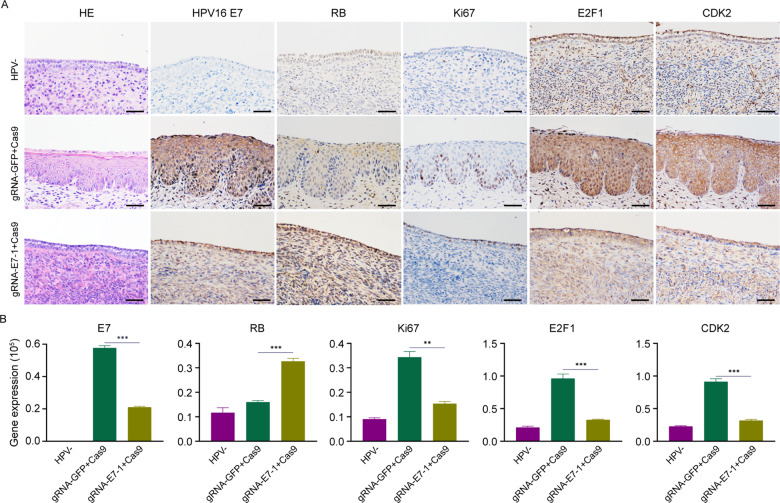
Histopathological and protein expression changes in cervical epithelia of K14-HPV16 transgenic mice treated with HPV16 E7 targeting CRISPR/Cas9. **A** Representative images of the HE staining and IHC staining of HPV16 E7, RB, Ki67, E2F1, and CDK2 in cervical epithelia of HPV-, gRNA-GFP + Cas9-treated, and gRNA-E7-1 + Cas9-treated mice. **B** Quantification of the protein expression of HPV16 E7, RB, Ki67, E2F1, and CDK2 in these 3 groups. **p < 0.01; ***p < 0.001. (*N* = 3 replications) Scale bars: 50 μm.

**Fig. 6:**
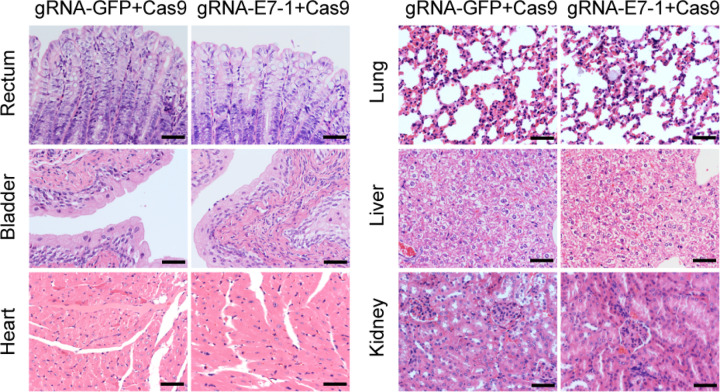
The conduction of regional plasmid transfection showed no influence on other organs. The HE staining of different organs in gRNA-E7-1 + Cas9 and gRNA-GFP + Cas9-treated K14-HPV16 transgenic mice. Scale bars: 50 μm.

We assigned the female K14-HPV16 positive mice randomly into 2 groups. The experimental group was treated with gRNA E7-1 + Cas9, while the control group was treated with gRNA-GFP + Cas9. FVB background K14-HPV16 negative mice of the same age was kept at the same time. We observed the HPV16 E7 DNA mutation in CRISPR/Cas9 treated mice after 24 days. DNA sequencing of the E7 gene showed deletions and point mutations after receiving treatment of CRISPR/Cas9, which displayed gene editing in this progression and was not observed in the control group. The examples above illustrated that the CRISPR/Cas9 system could induce DNA DSB and be repaired through the NHEJ repair way (Fig. 4D). After that, we continued to explore the pathological change of these 2 groups. HE staining showed that the gRNA E7-1 + Cas9-treated group could reverse the malignant phenotype of cervical epithelia in a gradual way, with normal nuclear and well-differentiated epithelia. IHC staining of HPV16 E7 decreased, with the RB expression restored, which indicated that the CRISPR/Cas9 system could inhibit the proliferation of HPV16 expression cells in vivo through the RB signaling pathway. The downstream expression of CDK2 and E2F1 also showed that the CRISPR/Cas9 system could induce cell cycle arrest, which was also illustrated by the downregulated expression of Ki67 (Fig. 5A). Quantifications of the protein expression of HPV16 E7, RB, Ki67, E2F1, and CDK2 in these groups were exhibited in Fig. 5B. To evaluate potential systemic side effects in treated mice, we got their organs other than the cervix for immunohistochemical staining and H&E staining at the endpoint. The IHC staining showed that there was no obvious Cas9 protein expression in these organs, and H&E staining showed that the treatment did not induce any significant morphological change in experimental and control groups (Fig. 6). In conclusion, the results showed that the CRISPR/Cas9 system could be a promising treatment method for cervical epithelial neoplasia in vivo.

## Discussion

The cervical cancer screening methods represented by Pap smear and high-risk HPV testing have advanced considerably over recent decades, which were significantly reduced cervical cancer deaths [21, 22] In current clinical treatment, surgical interventions were recommended for CIN2 + patients. However, it could introduce side effects such as infection and bleed, and there is also a potential impact on women’s pregnancy in the long term [23, 24]. Drug-based treatment might avoid these potential risks associated with surgery. In addition, there is no efficient treatment for persistent HPV infection patients until now. For these patients, surgery seems like overtreatment, however, regular follow-up will also cause socioeconomic burden and psychological burden of patients. In the primary prevention approach of cervical cancer, the HPV vaccine is ineffective for patients who have already been HPV infected [25]. Besides, the cost of the vaccination also limits its promotion especially in low-income and middle-income countries [26, 27]. Therefore, we suspect that even in the development of the HPV vaccine, there might still be a large number of HPV-infected people for a long time. In our study, we observed the designed CRISPR/Cas9 system had a favorable effect on cervical pre-cancer, and we considered it could be a novel therapeutic strategy to the existing treatment of HPV-related cervical lesions.

We used the S12 cell line derived from the low-grade cervical lesion and K14-HPV16 mice in vivo/in vitro [28, 29], which were representative cervical precancerous models. For K14-HPV16 mice, previous studies have observed that the premalignant stages of squamous carcinogenesis in the K14-HPV16 mice are highly similar to that of humans [29], making it an ideal model for cervical precancerous lesions. Through in vivo experiments, Hu et al. proved that intravaginally regional application of TALENs is an effective method of administration [10]. Following a period of administration of the CRISPR/Cas9 plasmid in K14-HPV16 mice, we found that the mouse cervical epithelium gradually reversed to histologically normal compared with the control group. Furthermore, we used IHC staining to evaluate the expression of the related proteins. In the CRISPR/Cas9-treated group, we found that the cleavage of the E7 gene resulted in downregulation of E7 protein expression, and the gradual restoration of expression of RB and its downstream targets E2F1 and CDK2, thus reversing the malignant phenotype of the cervix in vivo. Our research indicated that among cervical cancer-related lesions, especially cervical pre-cancerous lesions, CRISPR/Cas9 has promising clinical prospects.

CRISPR/Cas9 has been considered to have potential advantages for many chronic pathogenic diseases caused by DNA viruses, which cannot be cured using available drugs [30–32]. Compared with ZFN and TALEN, we observed that CRISPR/Cas9 also has significant growth-inhibitory and apoptotic effects on cervical cancer and cervical pre-cancer cell lines and inhibited the tumor formation of the S12 cell line in nude mice. In fact, the target sites of these 3 gene-editing tools were not exactly the same point; thus, the comparison between them also has its limits. However, the incomparably fast design process, high scalability, and affordability make CRISPR/Cas9 an ideal gene-editing tool compared with ZFN and TALEN.

The off-target effect and safety of CRISPR/Cas9 have always been the focus of our concern. Through in vivo and in vitro experiments, we observed that CRISPR/Cas9 could cause significant suppression of colony formation and cell growth and apoptosis of HPV16-positive cell lines S12 and SiHa. In contrast, CRISPR/Cas9 did not affect the growth of HPV18-positive cell line HeLa and HPV-negative C33A cells, demonstrating the specificity of HPV16-E7-targeted CRISPR/Cas9. Also, in the past, systemic injection was used in the application of CRISPR/Cas9, which might increase the risk of side effects. In our study, the administration of CRISPR/Cas9 was concentrated on the targeted site to ensure the therapeutic usefulness and lowest systemic side effects.

A recent study showed that during the infectious period, the HPV16 genome shared many amino acid-changing variants, while E7 protein was genetically conserved [17]. In addition, HPV16 E7 is considered to be a single oncoprotein that could cause cervical cancer in the animal model and immortalize human keratinocytes in vitro [33, 34]. This made HPV16 E7 an ideal target for the therapy of HPV16-induced cervical cancer, which means that our CRISPR/Cas9 system targeted for HPV16 E7 might have promising clinical applications. In addition, the HPV16 E6 gene is also a good candidate cleavage site for gene therapy, which has obtained promising results in vitro and in nude mouse models [8, 12]. We have also considered expanding the CRISPR/Cas9 cleavage sites of HPV16 in a follow-up study.

In our study, we noticed some other gene-editing tools showed similar efficiency with CRISPR/Cas9 in several HPV-positive cell lines. However, both ZFN and TALEN are modular proteins including the adaptable DNA binding domain fused with FokI, which is a complex process for designing these tools to target a novel DNA sequence. In contrast, the CRISPR/Cas9 system is a relatively simple designed system to overcome this shortage [35, 36]. Besides, the shRNA mainly targeted transcription products of HPV16 oncogene, which means it has a limited impact on the HPV DNA which was integrated into human cells, suggesting that this approach might only have a temporary effect. Furthermore, by designing multiple sgRNAs that target different genomic sequences, CRISPR/Cas9 system could editing multiple genes simultaneously, which could greatly enhance the therapeutic potential of this technology [37, 38]. Thus, we believe that although in some cancer cell lines, the CRISPR/Cas9 system and other techniques showed similar efficiency, this is still a promising tool for the therapy of cervical lesions.

This study still has some limitations. Our goal was to apply the designed HPV16 E7-targeted CRISPR/Cas9 system to clinical patients, but many factors still need to be taken into consideration, such as the vaginal fluid and pH value, which need to be confirmed by further experiments. In addition, the comprehensive analysis of host immune response and assessment of long-term effects were insufficient in our study. Three kinds of gene editing tools target different HPV16 E7 cleavage sites, and further experiments are needed to confirm their therapeutic effects. In this experiment, we focused on the HPV16 E7 protein, which may not explore the impact of other cleavage sites of HPV16. In future studies, we also hope to continue to design CRISPR/Cas9 systems for other cutting sites of HPV and explore their effect. Previous research also indicated that HPV integration would affect the local chromosome architecture of host cells, which could influence the progression of cervical carcinogenesis other than HPV oncoprotein [39]. Besides, the application of the CRISPR/Cas9 system could impact the three-dimensional (3D) genome structure of DNA [40]. We suspected the double-strand DNA breaks induced by our CRISPR/Cas9 system might have other potential mechanisms causing the death of target cells, which will need further validation. In addition, we would like to expand the number of treated mice in the follow-up study to further ensure the efficiency and safety of our CRISPR/Cas9 system.

In conclusion, we have demonstrated that CRISPR/Cas9 targeting HPV16 E7 can effectively reduce the expression of E7 protein in vivo/in vitro and have potential treatment effects on HPV-related cervical cancer and precancerous lesions.
